# Current Perspectives on Introgression Breeding in Food Legumes

**DOI:** 10.3389/fpls.2020.589189

**Published:** 2021-01-21

**Authors:** Aditya Pratap, Arpita Das, Shiv Kumar, Sanjeev Gupta

**Affiliations:** ^1^ICAR-Indian Institute of Pulses Research, Kanpur, India; ^2^Bidhan Chandra Krishi Viswavidyalaya, Mohanpur, India; ^3^International Center for Agricultural Research in the Dry Areas (ICARDA), Rabat Office, Rabat, Morocco

**Keywords:** pre-breeding, distant hybridization, food legumes, breeding populations, introgression line

## Abstract

Food legumes are important for defeating malnutrition and sustaining agri-food systems globally. Breeding efforts in legume crops have been largely confined to the exploitation of genetic variation available within the primary genepool, resulting in narrow genetic base. Introgression as a breeding scheme has been remarkably successful for an array of inheritance and molecular studies in food legumes. Crop wild relatives (CWRs), landraces, and exotic germplasm offer great potential for introgression of novel variation not only to widen the genetic base of the elite genepool for continuous incremental gains over breeding cycles but also to discover the cryptic genetic variation hitherto unexpressed. CWRs also harbor positive quantitative trait loci (QTLs) for improving agronomic traits. However, for transferring polygenic traits, “specialized population concept” has been advocated for transferring QTLs from CWR into elite backgrounds. Recently, introgression breeding has been successful in developing improved cultivars in chickpea (*Cicer arietinum*), pigeonpea (*Cajanus cajan*), peanut (*Arachis hypogaea*), lentil (*Lens culinaris*), mungbean (*Vigna radiata*), urdbean (*Vigna mungo*), and common bean (*Phaseolus vulgaris*). Successful examples indicated that the usable genetic variation could be exploited by unleashing new gene recombination and hidden variability even in late filial generations. In mungbean alone, distant hybridization has been deployed to develop seven improved commercial cultivars, whereas in urdbean, three such cultivars have been reported. Similarly, in chickpea, three superior cultivars have been developed from crosses between *C. arietinum* and *Cicer reticulatum*. Pigeonpea has benefited the most where different cytoplasmic male sterility genes have been transferred from CWRs, whereas a number of disease-resistant germplasm have also been developed in *Phaseolus*. As vertical gene transfer has resulted in most of the useful gene introgressions of practical importance in food legumes, the horizontal gene transfer through transgenic technology, somatic hybridization, and, more recently, intragenesis also offer promise. The gains through introgression breeding are significant and underline the need of bringing it in the purview of mainstream breeding while deploying tools and techniques to increase the recombination rate in wide crosses and reduce the linkage drag. The resurgence of interest in introgression breeding needs to be capitalized for development of commercial food legume cultivars.

## Introduction

Deployment of plant breeding tools has been successful for bolstering crop productivity, harmonizing crop phenology, enhancing nutritional quality, and developing resistance to multiple stresses. This became possible with identification of new combinations of genes and construction of superior populations possessing desirable novel characteristics, which have been exploited for human welfare (Anderson, 1949; Arnold, 1992). Although there are numerous examples for purposeful introgression of advantageous traits into crop varieties as a part of regular plant breeding programs, the extent and impact of either natural or farmer-aided introgression are yet to be ascertained (Jarvis and Hodgkin, 1998, 1999). With almost 20,000 species, legumes are the members of the Fabaceae/Leguminosae, the third largest family of the higher plants, which are ubiquitously present all over the temperate and tropical parts of the world (Polhill and Raven, 1981). Food legumes are important to human and animal life and occupy an important place in the global food supply chain, as well as sustainable agricultural production systems. With high protein content and 15 essential minerals, these are indispensable constituents of the cereal-based vegetarian diets and are grown traditionally with cereals, oilseeds, sugarcane, etc. Food legumes have prominent biological features and an inherent capability to fix atmospheric nitrogen owing to the presence of symbiotic association with *Rhizobium* bacteria in root nodules. Therefore, these crops become an indispensable part of the sustainable agricultsure strategy throughout the world (Chaturvedi et al., 2011).

A quest is on for the search of genes that can impart resistance to biotic and abiotic stresses in different food legumes, as well as to improve the physical and nutritional qualities of grains. The ever-changing climatic conditions led to emergence of new insect-pests and diseases and their biotypes and races, which are becoming a major threat limiting crop production and productivity (Chakraborty and Newton, 2011; Gautam et al., 2013). Broadening the genetic base will provide the needed armor to legume crops against these emerging challenges under climate change. The crop wild relatives (CWRs) are known to possess useful alien alleles and cryptic genetic variation, which are introgressed and expressed in cultivated genepool only when a systematic breeding scheme is put in place (Doyle, 1988; Tanksley and McCouch, 1997; Gupta and Singh, 2009; Pratap and Gupta, 2009). Recent advances in breeding and genomic tools and techniques provide an opportunity to introgress useful alleles left behind in the secondary and tertiary genepool into the elite background useful for legumes breeders. This review illustrates factors affecting wild gene introgression, population development, and success resorting wild gene introgression in cultivated food legumes.

The alien gene transfer in a crop species is paramount when the breeding value of the parental genepool no longer responds to selection, resulting in slow or no genetic gain. Conventionally, it is easier to manipulate desirable genes present within a crop species compared to the alien genes from distant relatives or exotic germplasm. This is because the gene transfer within a species is comparatively easy as there are no crossing barriers, and also it is largely free from linkage drags of unwanted traits. In food legumes, ∼3,700 improved varieties with narrow genetic base form the present varietal portfolio (Kumar et al., 2020), resulting in the genetic uniformity in farmers’ fields. These varieties have been developed by the repeated use of a handful of elite germplasm from the primary genepool and therefore resulted in narrow genetic base and limited genetic buffers (Kumar et al., 2004). Introgression of alien genes from CWR offers a viable option to diversify and widen the genetic base of legume varieties, which provide insulation against the vagaries, as well as scope for continuous genetic gains over many breeding cycles (Kumar et al., 2009).

The horizontal gene transfer from wild species and even across different genera has played a significant role in the evolution of eukaryotic genomes (Bock, 2009) as wild species have evolved through different degrees of selection pressure exerted by environmental forces and biotic agents over a long period of time. As a result, these species have acquired many useful genes/alleles imparting adaptation to environmental cues such as extreme temperature, drought, waterlogging, salinity, and mineral toxicity, as well as biotic factors such as diseases, insect-pests, parasitic weeds, etc. Thus, hybridizing wild species with elite germplasm following a proper breeding scheme offers scope for the generation of multitude of pre-bred lines with novel recombination, which can further be utilized in the mainstream breeding for continuous accelerated genetic gains.

## Genetic Bottlenecks and Germplasm Redundancy

During evolution and domestication, wild progenitors have graduated to the cultivated forms passing through various genetic modifications and acquiring a combination of traits referred as “domestication syndrome.” Nevertheless, the persistence of these species in nature for a long time, largely remaining unattended, might have led to disappearance of many genes/alleles responsible for input response and higher grain yield in legume crops (Jain, 1975). Further, only limited samples of the accessions representing the narrow genetic base of the total diversity might have been brought to the center of domestication leading to the “founder effect” (Ladizinsky, 1985). The history of food legumes matches with human civilization while their evolution took place throughout many different regions of the world (Pratap and Kumar, 2011). However, keeping in view that many food legumes now have their major production base away from the actual center of diversity and also that during their domestication limited sampling might have narrowed down their genetic base, these crops might have started their domestication journey with the “founder effect.” For example, limited genetic diversity is reported in soybean outside its center of origin (Shoemaker, 1986; Pratap et al., 2012). Likewise, *Phaseolus*, chickpea, lentil, and pigeonpea also witnessed this bottleneck during their domestication.

While large germplasm repository of food legumes is preserved in different genebanks across the globe, mining of genetic diversity for use in mainstream breeding remains limited because of the paucity of information on economic traits and the nature of diversity itself (Kumar et al., 2007). This becomes much more alarming when we consider the use of exotic and unadapted germplasm in breeding programs. Further, the large size of germplasm collection, breeders’ preference for elite × elite crosses due to obvious advantages of their adaptability to local conditions, presence of cryptic genetic variation, and the linkage drag associated with transferring genes from wild relatives are other factors associated with restricted use of germplasm (Sharma et al., 2016).

Linkage drag is one of the major apprehensions while utilizing exotic and wild species in genetic amelioration of food legumes. In most of the cases, undesirable linkages hinder the transfer of desirable traits into cultivated backgrounds, and breaking such linkages needs dedicated efforts with a larger population and an efficient selection pressure. To overcome the problem of linkage drag, an additional generation of crossing among progenies prior to the selection or recurrent selection program over several generations is recommended. Nonetheless, it is now possible to recover or transfer into the elite germplasm the favorable alleles that were inadvertently left behind during the process of domestication. This can be done more efficiently by deploying molecular maps and integrative quantitative trait locus (QTL) analysis (for details, see Chamarthi et al., 2011) either through constructing introgression libraries that are made up of several introgression lines (ILs) or utilizing advanced-backcross QTL (AB-QTL) analysis. Introgression libraries can be constructed by crossing cultivated parent with wild donor followed by three to four times backcrossing of F_1_ with cultivated parent (Kumar et al., 2011). In the past, attempts have been made to develop such libraries in soybean using *Glycine soja*, a wild species (Concibido et al., 2003), and from synthetic tetraploids in peanut (Fonceka et al., 2012). The AB-QTL approach also deploys repeated backcrossing involving elite parent and wild accession with an aim to reduce the number and size of the donor segment transferred through alien introgression. The ultimate objective here is to minimize the effect of linkage drag in such crosses, and advanced backcrossed populations thus derived are further subjected to QTL analysis to identify desirable genes/QTL. Common bean and soybean are the best examples where this approach has been used successfully (Blair et al., 2003; Chaky et al., 2003).

## Breeding Populations for Gene Introgression

**FIGURE 1:**
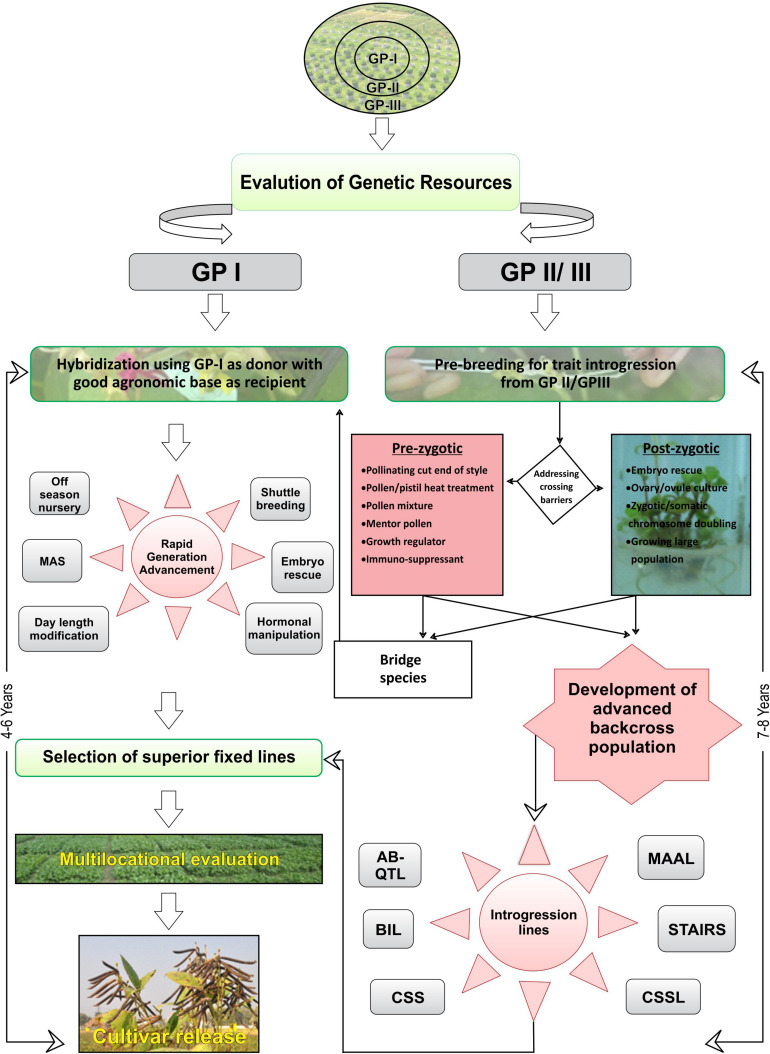
Scheme for gene introgression for improvement of food legumes. **Left**, Hybridization using GP-I as donor with good agronomic base as recipient. **Right**, Pre-breeding for trait introgression using GP-II and/or GP-III.

Crop wild relatives are valuable source of novel and cryptic variation for broadening the genetic base of cultivated genepool (Dwivedi et al., 2005; Pratap et al., 2014). CWRs also harbor superior QTLs for improving agronomic and yield attributing traits. However, currently available approaches for introgression are not suitable for polygenic traits because of selection bias against the alien alleles. Moreover, penetrance and expressivity of alien genes and traits when introgressed in the cultivated background are often incomplete and limited, resulting in poor genetic gains. As a result, breeding for introgression of QTLs from CWR to elite background is avoided, and emphasis has been laid upon transfer of oligogenic traits governing stress resistance mostly. Nevertheless, with the advancement in genomic tools and techniques, it has become feasible to identify and target selection for major QTLs from CWRs. For QTL analysis, mostly balanced populations (F_2_, BC_1_) have been utilized previously where alleles of both wild and elite populations are available in the same frequency, although these populations are easy to develop but are characterized by several drawbacks. Balanced populations have the most complete genetic construction and only allow for analyzing both dominant and additive effects (Wang and Chee, 2010). These populations are temporary and highly heterozygous; thus, it is difficult to use them in replicated yield trials because in every generation of either selfing or backcrossing, the genetic constitution of these populations would change. Moreover, undesirable QTLs from the unadapted wild background could lead to the linkage drag. Further, during the transfer of QTLs, epistatic component augments the complication because it is difficult to detect through statistical inference, often sensitive to environments, is difficult to manipulate, and is likely to be present in balanced populations (Bernardo, 2010). To overcome these difficulties, “specialized population concept” has been advocated for transferring QTLs from CWR into elite backgrounds. For details, please see Figure 1 The breeding population developed through different methods of gene introgression has been described in Table 1.

**TABLE 1:** Examples of AB-QTL analysis for wild QTL introgression in legumes.

Crop	Cross	Population	No. of lines	Marker systems	Trait	References
Pigeonpea	ICPW 68 × ICPL 85010	BC_4_F_12_	138	*–*	*Phytophthora drechsleri* resistance	Mallikarjuna et al., 2011
	ICPL 87119 × ICPW 12	BC_2_F_7_	149			
Chickpea	ICC 4958 × (ICC 17264 × IG 69978)	BC_2_F_4_	1,500	*–*	Agronomic traits	Sharma et al., 2017
	ICCV 95311 × (IG 72933 × ICC 20192)	BC_2_F_3_	2,000			
	EC556270 × GPF2	BC_2_F_3_	52	SSR	Agronomic traits	Bhavyasree et al., 2018
	ILWC21 × GPF2					
Field pea	*P. sativum* cv. Pennant × ATC113 (*P. fulvum*)	BC_1_F_3_	72	*–*	Resistance against Pea weevil	Aryamanesh et al., 2012
Common bean	ICA Cerinza (Andean) × G24404 (Colombian)	BC_2_F_3__:__5_	157	SCAR, SSR	Agronomic traits	Blair et al., 2006
	OR 91G (snap bean) × PI 255956 (runner bean)	BC_2_F_4_	115	AFLP, SSR	Resistance against White mold	Haggard, 2007
	Cerinza × G10022	BC_2_F_2__:__5_	138	SSR	Agronomic traits, Fe and Zn	Blair et al., 2012
Peanut	ICGV 91114 × ISATGR 1212	BC_2_F_9_	416	DArT	Agronomic traits, as well as biotic stresses	Mallikarjuna et al., 2012
	ICGV 87846 × ISATGR 265-5A	BC_2_F_9_	579			
	ICGV 87846 × ISATGR 278-18	BC_2_F_8_	250			
	TMV 2 × ISATGR 121250	BC_2_F_8_	686			
	Florunner × TxAG-6	BC_3_F_6_	90	SSR, RFLP	Oil quality	Wilson et al., 2017
	Florunner × TxAG-6	BC_3_F_6_	233	RFLP	Resistance to root knot nematode	Burow et al., 2014
	Fleur11 × (*A*. *ipaensis* KG30076 × *A*. *duranensis V14167*) ^4x^	BC_2_F_1_	87: BC_3_F_1_ and 55: BC_2_F_2_	SSR	Drought resistance	Fonceka et al., 2012

## Advanced-Backcross QTL

Advanced-backcross QTL strategy was proposed by Tanksley and Nelson (1996) for concurrently mining and transferring positive QTLs from CWR into elite genepool. It is a kind of inbred backcrossing for transferring complex trait from unadapted genepool to the elite background (Sullivan and Bliss, 1983). In this methodology, QTL analysis is deferred until advanced (BC_2_ or BC_3_ and so on) generations. This is because in early generation the effects of beneficial QTLs often remain unrecognized because of the presence of epistatic interactions between favorable QTLs and other genes from the donor parent, which might be resolved in later generations, thus allowing possible silencing of the measured QTL effects (Pillen et al., 2003). The common segregating populations (F_2_, BC_1_, RIL, and DH) generally utilized for QTL analysis have some major drawbacks when involving wild species in introgression program. First, these populations represent a large segment of genes from wild parent, and the QTLs with small effects remain unseen. Second, with these populations, the discovery and further introgression of QTLs through subsequent backcrossing or intercrossing becomes a two-step process, thus becoming a time-consuming affair with mere chance of utilizing QTL information to develop superior cultivar (Tanksley and Nelson, 1996). In AB-QTL strategy, the discovery and further transfer of positive QTLs from unadapted background to elite pool are a single-step process where QTL analysis is performed in later generations to facilitate sound statistical power for detection of QTLs with small effect.

The application of AB-QTL strategy has been tested in several food legumes and recommended as a useful crop breeding venture. An attempt was made to transfer QTLs conferring yield attributing traits from two accessions of *Cicer reticulatum*, “EC556270” and “ILWC21” into *Cicer arietinum* cv. GPF2 by attempting two cross combinations. The respective BC_2_F_3_ population was tested for yield attributing traits for confirmation of introgression of productivity traits into elite chickpea cultivar (Bhavyasree et al., 2018). Field pea weevil, *Bruchus pisorum*, is a severe menace in cultivated field pea (*Pisum sativum*). An attempt was made to develop AB population (BC_2_F_6_) by involving *Pisum fulvum* accession “ATC113” as a resistant donor and susceptible *P. sativum* cv. Pennant as recipient (Aryamanesh et al., 2012). Wild beans are very diverse and useful source for enriching genetic variation of cultivated beans with low diversity. AB population (BC_2_F_3__:__5_) was developed in common bean involving a cross between large red-seeded commercial Columbian variety, “ICA Cerinza” as recurrent parent and wild accession “G24404” for detecting QTL toward improvement of agronomic performance. This strategy uncovered 13 QTLs for plant height, yield, and yield-attributing traits along with detection of a QTL for seed size from the wild parent (Blair et al., 2006). Another attempt has been made in common bean to transfer resistance against white mold, caused by *Sclerotinia sclerotiorum*. AB-QTL strategy has been undertaken to identify and transfer QTL conferring resistance to white mold into an interspecific cross of *Phaseolus vulgaris* cv. OR91G and *Phaseolus coccineus* cv. PI255956. A population of 115 BC_2_F_4_ lines were developed and genotyped using amplified fragment length polymorphism (AFLP) and simple sequence repeat (SSR) markers and screened under greenhouse for phenotypic scoring (Haggard, 2007). Wild common beans accumulate high minerals but are not commercially acceptable because of small seed size. AB population was developed for improving the mineral status of the Andean variety “Cerinza,” a large red seeded bush bean cultivar with wild genotype “G10022.” The BC_2_F_3__:__5_ ILs derived from this cross combination were subjected to multilocation yield trial for contemplating the role of genotype × environment interaction toward the expression of Fe and Zn content in AB population (Blair et al., 2012). The result from the study confirmed that the AB-QTL method was effective for identifying the QTL controlling Fe and Zn content, as well as their transfer into elite background and further evaluation.

Therefore, it is evident that the AB-QTL approach has been successfully applied in numerous legume species for harnessing the favorable alleles from wild into elite background, although in many other crops, *viz*., mungbean, urdbean, *Lathyrus*, and lentil, this approach is yet to be employed to explore its advantages. This strategy has paved the way for identifying more QTLs, precise measurement of the effect of individual loci, and their transfer into the cultivated background. Additionally, the formation of AB-NIL would facilitate further genetic dissection of QTLs and subsequently the map-based cloning of the underlying genes, thus opening a new vista for other legumes also.

## Introgression Lines

Introgression lines are specialized populations derived through advanced backcrossing, which are nearly isogenic to recurrent parent and contain only a small fraction from donor parent (Eshed and Zamir, 1994; Tian et al., 2006). These populations are much more efficient for QTL identification and fine mapping followed by studying QTL × environment interaction due to their homozygous nature as compared to the conventional populations (Wehrhahn and Allard, 1965; Eshed and Zamir, 1995; Yin et al., 2016). The major drawback of ILs is the time taken for their development (Tanksley et al., 1989; Hospital et al., 1992; Hospital and Charcosset, 1997) and high cost for marker evaluation (Ali et al., 2010). However, with the availability of densely saturated marker systems in some legumes such as chickpea, pigeonpea, soybean, peanut, etc., the foreground as well as background selection become easier. In addition, marker-based selection facilitates detection of non-target introgression in early generation, as well as further elimination from the recipient background for speeding up IL recovery (Frisch and Melchinger, 2005).

The critical factors for reducing the problem of linkage drag in backcross population through marker-assisted selection are the tightness of the linkage between the introgressed genes and the flanking markers and the size of the population, as well as the total duration of the backcross scheme (Hospital, 2001) besides the size of the segment to be transferred. Theoretical explanation given by Hospital (2001) nicely pointed out that presence of functional markers within the genes to be introgressed or tightly linked flanking markers along with three to five generations of backcrossing would be cost-effective to minimize the length of the donor segment. In peanut, pyramiding of nematode resistance and the trait governing high oleic:linoleic acid has been introgressed successfully to develop improved Tifguard variety “Tifguard High O/L” through tightly linked cleaved amplified polymorphic sequences and SSR markers with less linkage drag problem (Chu et al., 2011). Another high oleic acid line was developed in peanut through marker-assisted introgression of two *FAD2* mutant alleles conferring high oleic acid from donor parent “SunOleic95R” into the background of “ICGV 06100” (Bera et al., 2019). In chickpea, two *Fusarium* wilt (FW) resistance ILs, namely, “Annigeri 1” and improved “JG 74,” have been developed through marker-assisted backcrossing using “WR 315” as the donor parent (Mannur et al., 2019). Foreground selection was done with TA59, TA96, TR19, TA27, and GA16 markers, whereas background selection was done using SSR markers. Likewise, Pratap et al. (2017) developed improved “Pusa 256” using “Vijay” as the donor parent using TA 37 and TA 110 as the markers for foreground selection. Two parallel marker-assisted introgression programs have been implemented to improve both FW, as well as *Ascochyta* blight (AB) resistance of “C 24” cultivar by introgressing resistant locus of race 1 of FW coupled with two QTL clusters for AB resistance (Varshney et al., 2014).

Besides conventional marker-assisted introgression approaches deployed in food legumes, various IL-based strategies have been recommended like backcross inbred lines (BILs), chromosome segment substitution lines (CSSLs), stepped aligned inbred recombinant strain (STAIRS), etc., for removing background noise and measuring yield associated traits precisely. BILs are characterized by small introgression of segment from donor parent and useful for reducing background noise from donor parent, as well as for mapping interspecific variation (Eshed and Zamir, 1994). In soybean, BIL populations have been developed for overcoming abiotic stresses by mining and introgressing useful QTLs from the donor parent. Water limiting situation is one of the main restraints for soybean production (Sinclair et al., 2007). Earlier reports confirmed that root length and absorption surface area along with root architecture are prime determinants for yield performance under variable moisture regime (Price et al., 2002; Manavalan et al., 2009). As variability regarding root architecture is limited in cultivated soybean, an attempt was made to explore the potential of exotic wild species for broadening the genetic base. BIL mapping population has been developed by crossing *Glycine max* cv. Dunbar (PI 552538) as a recipient with a wild soybean accession “PI326582A” of *G*. *soja*. BILs have been created to minimize the magnitude of gene introgression from the wild soybean parent “PI326582A” by allowing two generations of backcrossing to produce 296 BC_2_F_4_._5_ progenies (Manavalan et al., 2015). Genetic linkage map was constructed by using SSR and SNP markers, resulting in the identification of a major QTL (Satt315-I locus) on chromosome 8 that governs root traits and shoot length. It has been observed that, sometimes, the same metabolic pathway governs different stresses in plants (Xiong and Yang, 2003), and it is mostly associated with overlapping QTLs. The reason for this genetic overlapping is due to pleiotropy and linkage disequilibrium (Zhang et al., 2012). In soybean, BIL mapping population was developed by crossing a Chinese variety, “Hongfeng 11” with an American variety, “Harosoy” for mapping QTLs related to drought and low-temperature tolerance during germination. Finally, 12 QTLs were detected that were correlated with drought and low-temperature tolerance and confirmed the mechanism of partial genetic overlap between drought and low-temperature tolerance in soybean (Zhang et al., 2012). This study further validated the effectiveness of using BILs for gene introgression, trait identification, QTL mapping, and gene cloning in legume.

## Chromosome Segment Substitution Line

Chromosome segment substitution lines are very robust population for QTL mapping or cloning and gene discovery, as well as for gene pyramiding (Tanksley and Nelson, 1996), and can be developed by deploying AB strategy subsequently by selfing and selection of backcross population with molecular markers. Selection of backcross population with markers leads to identification of individuals carrying the introgressed gene(s) of interest along each chromosome. CSSLs generally exclude non-targeted portion from the donor, which can create background noise due to epistatic interaction (Ali et al., 2010). In CSSLs, each line carries a single defined chromosomal section from the wild donor into the recipient background, unlike BILs, where each line carries several homozygous introgressed segments from donor parent. Unlike BIL, CSSL libraries have been developed to recover the whole genome of donor parent (Ali et al., 2010). These populations can be compared with the individual IL or recurrent parent for finding out significant differences between them. CSSLs are also useful populations for controlling allelic variation and facilitate “breeding by design” (Peleman and van der Voort, 2003; Wei et al., 2010).

This strategy has been used in many crops including wheat (Liu et al., 2006), tomato (Monforte and Tanksley, 2000), rice (Bian et al., 2010), maize (Wang et al., 2007), cotton (Wang et al., 2008), and barley (Matus et al., 2003) for gene discovery and map-based cloning and opens a new vista for exploring the potential of CSSL population in legumes for detection of genes or QTL explicitly, as well as their pyramiding into elite background. Cultivated soybean (*G. max*) is domesticated from wild *G. soja* (Broich and Palmer, 1980), which harbors useful genes governing large number of pods, richness in protein, adaptability to various biotic and abiotic factors, etc. Previous studies confirmed the versatility of *G*. *soja* as a useful donor for enriching the genetic diversity of cultivated soybean (Concibido et al., 2003; Li et al., 2008). The problem of linkage drag often circumvents the useful introgression process. Keeping these in mind, attempt has been made to construct CSSL population consisting of 151 lines by involving *G*. *max* cv. NN1138-2 as female and *G*. *soja* cv. N24852 as male. Polymorphic SSR markers between the parents were deployed for marker assisted selection (MAS) for easy recovery of CSSL. In this study, four QTLs related with plant height, as well as node numbers per plant, have been identified (Wang et al., 2013). The same CSSL population was used for mining and fine mapping of QTLs underlying seed quality traits including size and shape, as well as other agronomic traits (Wang et al., 2012, 2013; He et al., 2014).

## Chromosome Substitution Strain

Another approach is the construction of chromosome substitution strain containing a large number of lines each carrying a homozygous chromosome with single crossover in such a way that the chromosome contains recurrent genotype at one end and donor genotype at the other end and known as single recombinant lines (SRLs). When the SRLs for each chromosome are sequentially stacked, they reveal a step-like progression, with each successive line having a little more donor chromosome, and constitute STAIRS libraries (Koumproglou et al., 2002). The concept was first applied in *Arabidopsis thaliana* for fine mapping of QTL. Although STAIRS has not yet been explored in legume crops, it is an effective strategy for comparison of genetic differences in the precise region of selected chromosome for QTL analysis, gene mining, and expression studies (Koumproglou et al., 2002). All these ILs can be maintained as an immortal representation in the form of “exotic library” for efficient detection and mapping of QTLs conferring agronomic traits (Zamir, 2001). This library is a permanent resource, which enables the researchers to explore over time and access the data generated for further use. The homozygous lines maintained in the library can be utilized as a parent for crossing with different tester lines to identify the chromosomal segments associated with heterosis. Development of an exotic library will immensely facilitate to counter the problem of linkage drag and precisely examine the phenotypic effect of QTL interaction for better insight into the epistatic effect (Eshed and Zamir, 1996). All these mapping populations along with genomic tools will be valuable for demonstrating the scope of introgression of desirable QTLs from CWR that was hitherto difficult to accomplish. The methodologies described can be extended to legume crops for harnessing the potential of CWRs for broadening the genepool through genomics-assisted genetic enhancement.

## Potential Wild Species for Alien Gene Transfer of Target Traits

Most food legumes and their wild relatives (CWRs) are diploid and self-pollinated in nature. Considerable variability exists in wild species for yield contributing traits including number of pods per plant, number of seeds per pod, and seed size, as well as nutritional traits and biotic and abiotic stress resistance. The success of alien gene transfer through distant hybridization generally depends on the ploidy level of the species, pollination behavior of the plant, nature, and direction of the cross and frequency of pollination, which are further influenced by the deployment of appropriate hybridization schemes (Pratap et al., 2015a). Efforts were made to identify potential wild accessions for alien gene introgression in different food legumes by several researchers (Table 2).

**TABLE 2:** Potential of wild species for alien gene transfer in food legumes.

Trait	Species	References
**Chickpea**		
*Ascochyta* blight	*C. bijugum* K.H. Rech.	Stamigna et al., 2000; Shah et al., 2005
	*C. echinospermum* P.H. Davis	Collard et al., 2003; Saeed and Darvishzadeh, 2017
	*C. judaicum* Boiss.	Stamigna et al., 2000; Shah et al., 2005; Saeed and Darvishzadeh, 2017
	*C. pinnatifidum* Jaub. & Sp.	Collard et al., 2001; Shah et al., 2005
	*C. reticulatum* Ladiz.	Shah et al., 2005; Saeed and Darvishzadeh, 2017
	*C. yamashitae* Kitamura	Shah et al., 2005
	*C. canariense* S. Guerra & Lewis	Kaiser et al., 1994
*Fusarium* wilt	*C. bijugum* K.H. Rech., *C. cuneatum* Hochst. Ex Rich, *C. echinospermum* P.H. Davis, *C. judaicum* Boiss., *C. pinnatifidum* Jaub. & Sp.	Singh K. B. et al., 1998
	*C. chorassanicum* (Bge) M. Pop.	Kaiser et al., 1994
	*C. reticulatum* Ladiz.	Infantino et al., 1996
	*C. canariense* S. Guerra & Lewis	Kaiser et al., 1994
*Botrytis* gray mold	*C. bijugum* K.H. Rech.	Isenegger et al., 2011
	*C. echinospermum* P.H. Davis	Knights et al., 2008
	*C. judaicum* Boiss., *C. reticulatum* Ladiz.	Pande et al., 2006
Root lesion nematode (*Pratylenchus thornei*)	*C. reticulatum* and *C. echinospermum*	Reen et al., 2019
Drought	*C. echinospermum* (ILWC 235), *C. oxyodon (C. oxyodon* L-4, L-9)	Saeed and Darvishzadeh, 2017
*Phytophthora* root rot	*C. echinospermum*	Amalraj et al., 2019
Drought	ICC7571	Kashiwagi et al., 2013
Terminal heat stress	ICC1205 and ICC15614	Devasirvatham et al., 2013
Drought	ICC14778	Krishnamurthy et al., 2013
Drought	*C. yamashitae*	Sharma and Upadhyaya, 2015; Sharma and Upadhyaya, 2019
Cold	*C. bijugum* K.H. Rech.	Toker, 2005
Drought		Imtiaz et al., 2011
Cold	*C. echinospermum* P.H. Davis	Toker, 2005; Saeed et al., 2010
	Drought and heat	Canci and Toker, 2009
Cold	*C. reticulatum* Ladiz	Singh K. B. et al., 1998; Toker, 2005; Saeed et al., 2010
Drought and heat		Canci and Toker, 2009; Imtiaz et al., 2011
Drought and heat	*C. anatolicum* Alef., *C. microphyllum* Benth., *C. montbretii* Jaub. et Sp., *C. oxydon* Boiss. et Hoh., *C. songaricum* Steph. ex DC.	Toker et al., 2007b
**Lentil**		
Anthracnose	*Lens ervoides, L. lamottei, L. nigricans*	Tullu et al., 2006
*Ascochyta* blight	*L. ervoides, L. culinaris* ssp. *orientalis, L. odemensis, L. nigricans, L. montbretti*	Bayaa et al., 1994; Tullu et al., 2010
*Fusarium* wilt	*L. culinaris* ssp. *orientalis, L. ervoides*	Gupta and Sharma, 2006; Singh et al., 2020
Powdery mildew	*L. culinaris* ssp. *orientalis, L. nigricans*	Gupta and Sharma, 2006
Rust	*L. culinaris* ssp. *orientalis, L. ervoides, L. nigricans, L. odemensis*	Gupta and Sharma, 2006
Drought	*L. odemensis, L. ervoides, L. nigricans*	Hamdi and Erskine, 1996; Gupta and Sharma, 2006
Cold	*L. culinaris* ssp. *orientalis*	Hamdi et al., 1996
*Orobanche*	*Lens ervoides, L. odemensis, L. orientalis*	Fernández-Aparicio et al., 2009
Bruchids	*L. culinaris Medikus* subsp. *orientalis, L. nigricans, L. lamottei*	Laserna-Ruiz et al., 2012
Rust and powdery mildew	*L. orientalis*	Singh et al., 2020
Powdery mildew and *Fusarium* wilt	*L. ervoides*	Singh et al., 2020
***Vigna species***		
Bruchid	*V. riukinensis, V. reflexo-pilosa*	Tomooka et al., 1992
	*V. radiata* var. *sublobata*	Miyagi et al., 2004
	*V. umbellata*	Somta et al., 2006
	*V. tenuicaulis*	Tomooka et al., 2000
	*V. nepalensis*	Somta et al., 2008
Powdery mildew	*V. stipulacea*	Tomooka et al., 2006
	*V. reflexo-pilosa* var. *glabra*	Egawa et al., 1996
Low trypsin inhibitor activity	*V. tenuicaulis*	Konarev et al.,(2002)
Chymotrypsin absent	*V. grandiflora*	Konarev et al.,(2002)
Heat	*V. aconitifolia*	Tomooka et al., 2001
	*V. riukinensis*	Egawa et al., 1999
Bean fly resistance	*V. reflexo-pilosa*	Egawa et al., 1996
Resistance to pod bug	*V. unguiculata* ssp. *dekindtiana TVNu 151*	Koona et al., 2002
Resistance to yellow mosaic virus	*V. radiata* var. *sublobata*	Reddy and Singh, 1990; Pal et al., 2000
	*V. umbellata, V. trilobata, V. mungo*	Pandiyan et al., 2008
Photothermo insensitivity	*V. umbellata, V. glabrescens*	Pratap et al., 2014
Soybean cyst nematode (*Heterodera glycines*)	*V. angularis*	Kushida et al., 2012
Salt stress	*V. luteola, V. marina, V. vexillata V. riukiuensis, V. trilobata, V. vexillata, V. marina* subsp. *oblonga, V. luteola*, and *V. marina*	Yoshida et al., 2020 Iseki et al., 2016
Salinity stress	Domesticated *V. unguiculata, V. vexillata*, wild *V. luteola, V. marina, V. nakashimae, V. riukiuensis, V. vexillata* var. *macrosperma.*	Van Zonneveld et al., 2020 Harouna et al., 2020
High temperature, salinity	*V. trilobata*	
Dry climate and salinity	*V. vexillata* var. *ovate*	
Dry and seasonally hot climate	*V. monantha, V. aconitifolia, V. aridicola, V. exilis*	
Resistance against Bruchid beetles	*V. radiata* var. *sublobata*	Schafleitner et al., 2016
Bruchids, *Cercospora* leaf spot, powdery mildew and MYMV	TCR 20	Tripathy et al., 2016
**Field pea**		
Drought tolerance	*P. fulvum*	Naim-Feil et al., 2017
TI1 and TI2 seed protease inhibitors	*P. sativum* subsp. *elatius*	Clemente et al., 2015
Pulse beetle (*Callosobruchus chinensis* L.)	*P. elatius* – AWP 442 and *P. fulvum* – AWP 600, AWP 601	Esen et al., 2019
Rust (*Uromyces* pisi.)	*P. fulvum*	Barilli et al., 2018
PSbMV virus (*Potyvirus*)	*P. fulvum*	Konečná et al., 2014
Powdery mildew	*P. fulvum*	Cobos et al., 2018
**Pigeonpea**		
Heat, drought	*C. acutifolius, C. cinereus, C. lanceolatus, C. latisepalus*	Khoury et al., 2015
Cold	*C. confertiflorus, C. mollis, C. platycarpus, C. trinervius*	
High precipitation, waterlogging, drought	*C. sericeus, C. lineatus*	
Heat, temperature variation/seasonality, cold	*C. platycarpus, C. scarabaeoides*	
Insect resistance – *Helicoverpa armigera*	*C. scarabaeoides* – IBS 3471	Ngugi-Dawit et al., 2020
*Helicoverpa* pod borer	*C. scarabaeoides, C. sericeus, C. lineatus, C. acutifolius*, and *C. platycarpus*	Saxena et al., 2018
Pod fly	*C. sericeus*	
Bruchids	*C. scarabaeoides, C. platycarpus*, and *C. acutifolius*	
Water logging	*C. acutifolius*	Hingane et al., 2015; Mallikarjuna et al., 2017
Antinutritional factors, high antioxidant potential	*C. scarabaeoides* (ICP15683/W15)	Sekhon et al., 2017
**Common bean**		
Nutritional composition and cooking characteristics	*Phaseolus acutifolius*	Porch et al., 2017
Abiotic stresses	*P. acutifolius*	Gujaria-Verma et al., 2016
Drought-tolerant	*P. acutifolius*	Mwale et al., 2020
Abiotic stresses – drought and subzero temperatures	*P. acutifolius* A. (Gray)	Souter et al., 2017
**Cow pea**		
Heat and salinity	*V. unguiculata* group *sesquipedalis*	Van Zonneveld et al., 2020
Aphid	Wild cowpea relative – line TVNu 1158	Boukar et al., 2019
**Peanut**		
Late leaf spot pathogen	*Arachis diogoi*	Kumar and Kirti, 2015
Drought tolerance	*A. duranensis*	Lílian et al., 2019

Chickpea is the most important cool season grain legume and offers tremendous opportunities for its genetic improvement through introgression breeding, especially concerning biotic and abiotic stresses. Of the eight annual species, only one wild species, *C. reticulatum*, is readily crossable with the cultivated chickpea (Kumar et al., 2003). The success of hybridization with the remaining annual wild *Cicer* species requires specialized techniques such as the application of growth hormones and embryo rescue techniques (Lulsdorf et al., 2005; Mallikarjuna and Jadhav, 2008). Among the biotic stresses, FW and AB cause maximum damage to the plant and lead to severe yield reduction. FW causes up to 100% yield losses (Sharma et al., 2004; Pratap et al., 2017). AB, caused by *Ascochyta rabiei* usually appears at the reproductive phase, and in severe cases, the entire plant dries up suddenly. Several accessions of *Cicer bijugum, Cicer echinospermum, Cicer judaicum, Cicer chorassanicum, Cicer pinnatifidum, C. reticulatum, Cicer cuneatum, Cicer yamashitae*, and *Cicer canariense* have shown high resistance to AB (Haware et al., 1992; Kaiser et al., 1994; Collard et al., 2001; Shah et al., 2005). Simultaneously, many of them also possessed a high degree of resistance to FW (Kaiser et al., 1994; Infantino et al., 1996; Singh K. B. et al., 1998; Collard et al., 2001; Shah et al., 2005; Pande et al., 2006). Some of the accessions belonging to *C. bijugum, C. echinospermum, C. judaicum*, and *C. reticulatum* were reported to be highly resistant to *Botrytis* gray mold (Pande et al., 2006; Knights et al., 2008; Isenegger et al., 2011; Coyne et al., 2020). Several chickpea CWRs have shown high tolerance to abiotic stresses. For example, tolerance to drought and heat stresses was reported in six *Cicer* species (Toker et al., 2007a; Canci and Toker, 2009; Imtiaz et al., 2011). Likewise, tolerance to cold was reported in *C. bijugum, C. echinospermum, C. pinnatifidum*, and *C. reticulatum* (Singh et al., 1990; Robertson et al., 1995; Singh K. B. et al., 1998; Toker, 2005; Saeed et al., 2010).

Among *Vigna* crops, the Asiatic *Vigna* have tremendous scope for improvement with respect to yield and yield attributes, biotic and abiotic resistance, and nutritional quality (For review, see Pratap et al., 2020). Accessions of *Vigna mungo* var. *silvestris* have been identified as a durable source of mungbean yellow mosaic virus (MYMV) resistance (Reddy and Singh, 1993). Likewise, variation for yield contributing traits and MYMV resistance was observed in *V. mungo* var. *silvestris, Vigna radiata* var. *sublobata* (Singh, 1990), *Vigna umbellata, Vigna trilobata*, and *V. mungo* (Nagaraj et al., 1981; Singh and Dikshit, 2002; Pratap et al., 2018a). “PLN15,” a wild accession of *V. radiata* var. *sublobata* possessed a high number of pods per plant and seeds per pod and therefore identified as a potential donor for these traits (Reddy and Singh, 1990). *V. trilobata* and *Vigna stipulacea* was proposed as the candidates for neodomestication for drought tolerance and disease and pest resistance, respectively, by Tomooka et al. (2014), whereas resistance to diseases and pests in both the species was also reported in several other reports (Nagaraj et al., 1981; Chandel et al., 1984; Tomooka et al., 2006; Pandiyan et al., 2008; Gore et al., 2019). Accession “TC1966” of *V. radiata* var. *sublobata* was reported to possess bruchid tolerance (Tomooka et al., 1992). Likewise, resistance to legume pod borers and pod-sucking bugs was reported in *Vigna vexillata* and *Vigna oblongifolia* (Fatokun, 1991). *V. mungo* var. *silvestris* was reported as immune to bruchids (Fujii et al., 1989; Dongre et al., 1996). Many accessions of ricebean (*V. umbellata*) show complete resistance to bruchids and therefore have been identified as useful donors for introgressing bruchid resistance into other *Vigna* species. Further, as it is a cultivated species, gene introgression from ricebean into mungbean and urdbean is comparatively easier. Dense hairs on different parts of the wild cowpea, *V. vexillata*, are reported to impart antixenosis to pod-sucking bugs and pod borer (Oghiakhe et al., 1992; Boukar et al., 2013). Likewise, strength and hardness of the pod wall are also considered to impart pod borer resistance (Oigiangbe et al., 2002). Other wild *Vigna* species with resistance to *Maruca vitrata* and the pod-sucking bugs include *Vigna unguiculata* ssp. *dekindtiana, Vigna luteola, Vigna oblongifolia*, and *Vigna reticulata*. Pratap et al. (2014) reported *V. umbellata* (accession IC251442) and *Vigna glabrescens* (accession IC251372) as photo- and thermo-period insensitive as these were able to flower and set pods at temperatures as high as 43.9°C and as low as 2.7°C.

Wild *Lens* species have emerged as a great reservoir of useful genes for traits of breeders’ interest including resistance to strategically important diseases, insect-pests, and plant parasitic weeds. A high degree of resistance was observed for *Stemphylium* blight in *Lens lamottei* followed by *Lens ervoides* (Podder et al., 2013). Similarly, some accessions of *Lens odemensis* possessed high resistance against *Sitona* weevil followed by *L. ervoides* (El-Bouhssini et al., 2008). Some of the wild accessions with combined resistance to AB and FW or anthracnose diseases have been identified for their use in lentil breeding programs (Bayaa et al., 1995; Tullu et al., 2006). Preliminary screening of *Lens* CWR has indicated drought tolerance in *Lens nigricans, L. odemensis*, and *L. ervoides* (Gupta and Sharma, 2006) and cold tolerance in *Lens culinaris* ssp. *orientalis* (Hamdi et al., 1996). Simultaneously, promising donors for yield traits, *viz*., 100-seed weight and pods per plant were observed in *L. lamottei* and *L. culinaris* ssp. *orientalis* (Gupta and Sharma, 2006). Earlier reports have indicated *L. ervoides* as a good source of alleles for plant architectural traits such as phenology, plant growth habit, and biomass besides seed traits (Tullu et al., 2011, 2013; Kumar et al., 2014). Based on the extensive evaluation of global wild *Lens* taxa representing 27 countries, Kumar et al. (2014) observed wide variation for yield attributing traits, as well as resistance to multiple diseases in *L. nigricans* and *L. ervoides*. Nutritional quality traits in wild *Lens* showed significant diversity not only for micronutrients (Sen Gupta et al., 2016; Kumar et al., 2018) but also for prebiotics, RFO, raffinose, and verbascose (Tahir et al., 2011).

## Gene Introgression in Food Legumes: Success

Wild gene introgression as a breeding strategy has been deployed successfully in food legumes for development of improved varieties, pre-bred lines, genetic stocks, mapping populations, and bridge species. Legumes such as chickpea, pigeonpea, lentil, mungbean, urdbean, and peanut have benefited from the wild gene introgression with successful examples of discovery, development, and deployment of useful traits in cultivated genepool. In chickpea, after the first report of successful interspecific crosses between *C. arietinum* and *C. reticulatum* (Ladizinsky and Adler, 1976), attempts have been made for crossing between *C. arietinum* and *C. echinospermum* (Singh and Ocampo, 1993; Pundir and Mengesha, 1995). *C. reticulatum* accession “ILWC119,” when involved in hybridization program, led to the development of “ILC10765” and “ILC10766,” two cyst nematode-resistant chickpea lines (Malhotra et al., 2002). Singh et al. (2005) and Ramgopal et al. (2012) utilized the diversity of *C. reticulatum* and *C. echinospermum* to transfer useful traits including tolerance to cold and resistance to diseases such as wilt, root rot, and *Botrytis* gray mold into cultivated chickpea. There are reports of successful interspecific crosses between *C. arietinum* and *C. judaicum* (Singh et al., 1999), *C. arietinum* and *C. cuneatum* (Singh and Singh, 1989), *C. arietinum* and *C. pinnatifidum* (Badami et al., 1997; Mallikarjuna and Jadhav, 2008), and *C. arietinum* and *C. bijugum* (Mallikarjuna et al., 2007). Successful introgression of useful genes into cultivated chickpea from these crosses has shown the transferability even from the cross-incompatible wild *Cicer* species.

Successful interspecific hybridization of *P. vulgaris* has been reported to a limited extent with the members of other wild *Phaseolus* species. Interspecific hybrids with *Phaseolus costaricensis* in the secondary genepool were reported by Singh et al. (1997), and consequently, VRW 32 was reported as the first white mold-resistant interspecific breeding line derived from *P. costaricensis.* Congruity backcrossing (CBC) involves recurrent backcrossing to each parent in alternate generations as opposed to the traditional recurrent backcrossing to a single recurrent parent and was first reported as a method to produce fertile intermediate hybrids between *Phaseolus acutifolius* and *P. vulgaris* (Haghighi and Ascher, 1988). This method allows substantial recombination between distant species, and new phenotypes can arise as a result of CBC (Anderson et al., 1996). Singh et al. (2009) reported the release of white mold-resistant “VCW 54” and “VCW 55” bean germplasm lines that were developed using CBC between the black bean cultivar “ICA Pijao” and the scarlet runner bean accession “G35172.” CBC has also been used to transfer traits from wild tepary species *Phaseolus parvifolius* into common bean (Singh S. P. et al., 1998).

Wilkinson (1983) reported a root rot-resistant line “Cornell 2114-12” derived from a cross between common bean and scarlet runner bean lines. Likewise, Miklas et al. (1999) developed the common bacterial blight-resistant bean germplasm lines “ICB-3,” “IBC-6,” “ICB-8,” and “ICB-10,” which were derived from an interspecific cross with scarlet runner bean. Beaver et al. (2012) released a bean germplasm line “PR0650-31,” which was derived from the cross BAT 93/PI 417662//VAX 6 using wild-type bean germplasm “PI 417662” collected from Jalisco, Mexico, and was resistant to web blight and common bacterial blight. Acosta-Gallegos et al. (2007) developed an inbred backcross population from a cross between G 24423, a wild bean accession from Colombia and “Negro Tacana,” a Mexican black bean cultivar. One Bc2F4:7 line from this population was later observed to produce >5,000 kg/ha seed in field trials.

Lentil CWRs have been evaluated extensively to discover and deploy traits of interest into cultivated species. These efforts have led to identification of extra early photoinsensitive (ILWL118 maturing in <90 days) and high micronutrient content (ILWL74 and ILWL80) germplasm. These CWRs have been used extensively in mainstream breeding, resulting in the development of short-duration biofortified pre-bred lines (Kumar et al., 2018). Wide crosses in lentil have also been mined for transgressive segregants for agronomically important traits (Kumar et al., 2011; 2014; Singh et al., 2013). More recently, hybridization of the cultivated lentil with *L. ervoides* using embryo rescue (Tullu et al., 2013) has been reported with successful transfer of resistance to *Orobanche crenata* and anthracnose in cultivated species (Fiala et al., 2009; Tullu et al., 2011). The International Center for Agricultural Research in the Dry Areas has successfully deployed *L. orientalis* and *L. ervoides* for introgression of resistance to key diseases, phenology, biofortification, plant habit, and other important agronomic traits toward the development of pre-bred lines. These pre-bred lines demonstrated >40% yield advantage over the best check (Bakaria) coupled with richness in micronutrients. These pre-bred lines can also fit well in short-season windows of 80–100 days (Kumar et al., 2020). These lines are currently under multilocation testing under the CWR project.

In pigeonpea, despite large visible genetic variation (Yang et al., 2006), the use of wild species in breeding programs has been rather limited to the development of cytoplasmic genic male sterility systems (Saxena et al., 2010). To date, seven cytoplasmic male sterility (CMS) systems have been reported (Saxena et al., 2010), and six of them have been developed from wild relatives belonging to the secondary genepool. The seventh system was developed utilizing *Cajanus platycarpus*, a member from the tertiary genepool (Saxena et al., 2010; Mallikarjuna et al., 2011). The A_1_ CMS system derived from *Cajanus sericeus* (Saxena et al., 1996) was not stable at low temperature (<10°C) as the male-sterile plants revert to male fertility (Saxena et al., 2005). However, the presence of pollen shedders in the female line and non-availability of good maintainers did not make it commercially viable for hybrid breeding. The A_2_ cytoplasm derived from *Cajanus scarabaeoides* was reported as highly stable (Tikka et al., 1997; Saxena and Kumar, 2003). Although this system is promising with respect to yield, inconsistency was observed in the fertility restoration over diverse environments, which reduced its acceptance for hybrid production. In the A_3_ system, the cytoplasm was derived from *Cajanus volubilis* (Wanjari et al., 1999). The A_4_ CMS system was developed from *Cajanus cajanifolius*, which is so far the best among different CMS systems developed. This CMS system has a good number of both maintainers and restorers. In A_5_ system, the cytoplasm of cultivated species of *Cajanus cajan* was placed along with nuclear genome of a CWR of pigeonpea, *Cajanus acutifolius* (Mallikarjuna and Saxena, 2005) while using *C. cajan* as the female parent. This system also exhibited perfect fertility restoration by cultivated accessions. The A_6_ cytoplasm was developed from *Cajanus lineatus* (A_6_) in 2002, from one naturally out-crossed plant with erect growth and different morphological traits. This CMS system was observed to be very stable (Saxena et al., 2010) showing perfect fertility restoration by cultivated accessions. The A_7_ cytoplasm derived from *C. platycarpus* (A_7_) produced good heterosis (Saxena et al., 2010). Four CMS lines, *viz*., “GT 288A,” “CMS 67A,” “ICRISAT CMS,” and “AKCMS 1A,” were developed from different wild sources *viz*., *C. sericeus, C. scarabaeoides*, and *C. volubilis* (Chauhan et al., 2008).

In *Vigna* species, mungbean × urdbean hybridization has been routinely practiced for mungbean and urdbean improvement programs, and the derivatives from these hybridizations exhibit many desirable features *viz*., resistance to vagaries, both biotic and abiotic, synchronous podding, and non-shattering pods (Pratap et al., 2019). Several traits, such as longer pods, increased seeds number (>10 seeds/pod), and erect plant type, have been transferred from mungbean to urdbean, whereas multiple clusters per peduncle and sympodial pod-bearing habit have been transferred from urdbean into mungbean (Gupta et al., 2004). Similarly, mungbean × ricebean and mungbean × *V. radiata* var. *sublobata* hybridization have also been practiced by many breeders, and progenies were derived which were resistant to MYMV (Verma and Brar, 1996). Singh et al. (2003) produced successful hybrids between *V. radiata* and *V. umbellata* with intermediate morphology and MYMV resistance. Several popular and widely adaptable cultivars have been developed as a result of wild gene introgression in both mungbean and urdbean (Table 3). These cultivars show wide adaptation, synchronous maturity, and improved plant architecture in addition to a high degree of resistance to MYMV. Recently, “IPM 312-20” and “Tripura Mung-1” have been developed as a result of mungbean × urdbean hybridization. Likewise, the resultants of mungbean × urdbean crosses were also used further to develop some of the most popular varieties of mungbean. For example, IPM 99-125 was used to develop the most popular pan India variety “IPM 205-7” (Virat) of mungbean (Pratap et al., 2013) which matures in 52–55 days and offers the farmers an excellent choice for cultivation during summer season. Earlier, “IPM 02-3” was also a highly preferred variety of mungbean by farmers (Singh et al., 2017).

**TABLE 3:** Commercial cultivars of mungbean and blackgram developed through wild gene introgression.

Crop/variety	Pedigree	Introgression
**Mungbean**		
Pant Mung-4	T 44 × UPU 2	*V. radiata* × *V. mungo*
HUM-1	BHUM 1 × Pant U-30	*V. radiata* × *V. mungo*
Meha	Pant Mung-2 × AMP 36	*V. radiata* × amphidiploid of (*V. radiata* × *V. mungo*)
Pant Moong-6	Pant Mung-2 × AMP-36	*V. radiata* × amphidiploid of (*V. radiata* × *V. mungo*)
IPM 312-20	IPM 3-1 × SPS 5	*V. radiata* × *V. mungo*
Tripura Mung 1 (TRCM 131)	IPM 99-125 × SPS 5	*V. radiata* × *V. mungo*
**Blackgram**		
Mash 118	Mungbean × urdbean	*V. radiata* × *V. mungo*
Vamban 7	Vamban-3 × *V. mungo* var. *silvestris*	*V. mungo* × *V. mungo* var. *silvestris*
VBN 6	VBN 1 × *V. mungo* var. *silvestris*	*V. mungo*
TU-40	TU 94-2 × *V. mungo* var. *silvestris*	*V. mungo*
VBG 04-008	Vamban 3 × *V. mungo* var. *silvestris*	*var. silvestris*

In urdbean, at least five commercial varieties have been developed and deployed using wild gene introgression. The first such variety was Mash 118 developed from an urdbean × mungbean cross in 2008. This was followed by the development of four more cultivars, *viz*., Vamban 7, VBN 6, TU 40, and VBG04-008 in 2011. Among these, VBG04-008 showed high tolerance to heat stress, making it most popular cultivar in heat-prone environments of South India. Interspecific crosses have also been attempted successfully between *V. umbellata* and its wild relatives. However, the success of crosses with respect to pod set differed with the combination of parents involved in the interspecific crosses (Chen et al., 1983; Bharathi et al., 2006).

## Epilogue

The narrow genetic base of the elite genepool of food legumes and resultant vulnerability of the existing varieties to climate vagaries and changing insect-pest and disease scenario warrants introgression of novel genes or alleles through hybridization and deployment of more diverse germplasm including exotic lines and CWRs in crop improvement programs. Food legumes being majorly grown by small and marginal farmers are more prone to fluctuations in the soil, water, and climate factors as compared to other crops due to limited resources at disposal of these farmers to counter these challenges. The slow process of natural evolution has been significantly replaced by human interventions of domestication, hybridization, and selection. The transformation of humans from food collector to food producer has witnessed the natural attempts of domestication to a planned and focused crop breeding, which has ultimately concluded into the modern “super domestication.” While at one side, this has ensured food and nutritional security to ever-increasing population, on the other side, it has narrowed down the genetic base of the cultivated genepool. Keeping this in view, there is a need to reorient legumes improvement programs in such a way that more diverse sources of yield contributing traits, resistance to stresses, both biotic and abiotic, and seed quality are involved in widening the genetic base of cultivated types. This requires trait discovery and deployment from CWR, exotic germplasm, and landraces in mainstream breeding programs. A huge repository of germplasm and CWR (>7 million germplasm accessions) of different crops is maintained together in more than 1,750 national and international genebanks. This includes >86,000 accessions of chickpea^1^, >57,000 of *Phaseolus* (Basavaraja et al., 2020), >43,000 of mungbean (Nair et al., 2013; Gayacharan et al., 2020), >13,500 of pigeonpea (Upadhyaya et al., 2011), >16,000 accessions of cowpea, and so on. Nonetheless, characterization and evaluation data on economically important traits are limited to a smaller set of cultivated genepool. The situation is still worse when it comes to CWRs, which needs to be addressed on priority. The genetic bottlenecks leading to narrowing down the genetic base of food legumes need to be recognized and efforts to be initiated through intensive pre-breeding programs. However, owing to pre- and post-fertilization barriers applicable to distant crosses, special tools and techniques need to be adopted. These include application of growth hormones, using mentor pollen technique, deployment of embryo rescue, and several other methods bypassing these barriers (Pratap et al., 2010, 2018b). Wild gene introgression has yielded dividends in some legume crops such as mungbean, urdbean, pigeonpea, chickpea, lentil, etc., either directly through development of commercial cultivars or indirectly through the development of breeding materials and male sterile lines helping in hybrid variety development. Nonetheless, the advantages gained are still far from the potential of gene introgression, and focused planning and implementation in this direction are needed. Development of ILs, NILs, and specialized experimental populations may help in unleashing the genetic and genomic potential of wild gene introgression in the improvement of cultivated food legumes. These populations when subjected to precise and high throughput phenotyping will provide fast and inexpensive genomic information (Pratap et al., 2015b). Wild genetic resources are enormous, opportunities are tremendous, and challenges are manifold. Thus, the need is to venture into the wild gene introgression approach as a long-term strategy with great patience and care.

## Author Contributions

All authors listed have made a substantial, direct and intellectual contribution to the work, and approved it for publication.

## Conflict of Interest

The authors declare that the research was conducted in the absence of any commercial or financial relationships that could be construed as a potential conflict of interest.

## Footnotes

1 www.croptrust.com
